# * AKT1* but not *AKT2* single nucleotide polymorphisms are associated with the risk of microscopic polyangiitis

**DOI:** 10.7717/peerj.20791

**Published:** 2026-02-16

**Authors:** Lizhen Li, Huifang Tan, You Peng, Liepeng Chu, Jing Yang, Wenlv Tang, Kui Tan, Shuangshuang Fu, Meili Huang, Meijun Xu, Jinlan Rao, Chao Xue, Yinyin Chen

**Affiliations:** 1 Department of Nephrology, Hunan Provincial People’s Hospital, the First-Affiliated Hospital of Hunan Normal University, Changsha, China; 2 Hunan Institute of Geriatric Medicine, Clinical Research Center for Geriatric Major Chronic Diseases in Hunan Province, Hunan Provincial People’s Hospital, the First-Affiliated Hospital of Hunan Normal University, Changsha, China; 3 Department of Nephrology, the Second Affiliated Hospital of Guangxi Medical University, Nanning City, China

**Keywords:** AKT1, ANCA-associated vasculitis, Microscopic polyangiitis, Single nucleotide polymorphism, Genetic susceptibility, AKT2

## Abstract

**Background:**

Microscopic polyangiitis (MPA), a severe antineutrophil cytoplasmic antibody associated vasculitis (ANCA-associated vasculitis, AAV), demonstrates strong clinical association with myeloperoxidase/perinuclear anti-neutrophilic cytoplasmic antibodies (MPO/P-ANCA). While genetic factors are known to contribute to MPA susceptibility, the potential roles of AKT signaling components remain incompletely characterized, with limited data available for AKT1 and even less for its homologous gene AKT2 in this specific disease context.

**Methods:**

This case-control analysis included 798 participants (202 MPA patients and 596 controls, the latter comprising 387 individuals from the 1,000 Genomes Project), with control groups pooled after confirmation of genetic homogeneity. Genotypes of seven single-nucleotidepolymorphisms (SNPs) (four in AKT1, three in AKT2) with divergent allele frequencies across populations were analyzed. Association analyses were conducted under multiple genetic models, with gene-level and set-based approaches employed to evaluate aggregate effects. Secondary analyses included haplotype reconstruction, SNP-SNP interaction testing, and functional characterization through expression quantitative trait locus (eQTL) mapping.

**Results:**

Specific AKT1 variants (rs2498786 and rs1130233) demonstrated significant associations with reduced MPA risk, particularly in P-ANCA-positive patients. Gene-level analyses revealed a strong association for the AKT1 gene set (OR = 0.884, *P* = 0.002) but not for AKT2. Haplotype analysis identified protective AKT1 haplotypes, while interaction testing revealed high-risk genotype combinations. eQTL analysis indicated that protective alleles correlate with enhanced AKT1 expression in immune-relevant tissues, suggesting a potential regulatory mechanism.

**Conclusions:**

AKT1 emerges from this study as a likely genetic contributor to MPA, with its influence potentially involving neutrophil regulatory functions and vascular maintenance. The consistent absence of association signals for AKT2 across all analytical approaches could be viewed as reinforcing the specificity of AKT1’s involvement. These insights help refine the genetic architecture of MPA and position AKT1 signaling as a candidate pathway for future therapeutic exploration.

## Introduction

Antineutrophil cytoplasmic antibody (ANCA) are a group of autoantibodies targeting neutrophil cytoplasmic antigens, and are classified into Proteinase 3/Cytoplasmic ANCA (PR3/C-ANCA) and MPO/P-ANCA based on their target antigens and immunofluorescence staining patterns (Bossuyt et al., 2020). ANCA-associated vasculitis is a group of autoimmune disorders characterized by necrotizing inflammation of small-to-medium-sized vessels, with diagnosis contingent upon ANCA serological testing and specific histopathological alterations (Kronbichler et al., 2024). As a core subtype within this disease spectrum, microscopic polyangiitis (MPA) is distinguished by the absence of granuloma formation; its strong association with MPO/P-ANCA not only serves as a diagnostic hallmark (Kronbichler et al., 2024; Guchelaar et al., 2021) but also helps elucidate molecular pathways underlying the pathophysiology of vasculitis at the level of pathogenetic mechanisms (Kitching et al., 2020). In addition to ANCA, genetic factors such as SNPs are also believed to contribute to the pathogenesis of MPA (Trivioli et al., 2022). Numerous studies have demonstrated that polymorphisms in the human leukocyte antigen (HLA) gene were associated with susceptibility to MPA. For example, the *HLA-DQB1* (rs1049072) allele was linked to MPA risk in the American population (Merkel et al., 2017), while *HLA-DQ* (rs5000634) was associated with MPA in the European population (Lyons et al., 2012). Additionally, the HLA-DRB1*09:01 and HLA-DQA1*03:02 alleles were identified as risk factors for MPA in the Japanese population (Kawasaki et al., 2023). Research has also established a connection between HLA gene polymorphisms and ANCA. Specifically, *HLA-DQA2* (rs3998159, rs7454108) and *HLA-DQB1* (rs1049072) were associated with MPO/P-ANCA in the American population (Merkel et al., 2017), whereas HLA-DQA1*03:02 and DQB1*03:03 were linked to MPO-AAV in the Chinese population (Wang et al., 2019). Beyond HLA genes, polymorphisms in other genes have also been implicated in MPA. For instance, *PTPN22* (rs2476601) (Cao et al., 2015; Martorana et al., 2012; Merkel et al., 2017) and *PTPN22* (rs6679677) (Merkel et al., 2017) were associated with MPA in the European population, and *FCGR3B* copy number variations (Fanciulli et al., 2007; Martorana et al., 2016) have been linked to MPA. Furthermore, the *TLR9* (3-SNP haplotype) was associated with both MPA and MPO/P-ANCA (Husmann et al., 2014; Wang et al., 2020), while *BACH2* (rs78275221) was found to correlate specifically with MPO/P-ANCA (Dahlqvist et al., 2022). Other genetic factors that may influence MPA risk include *CTLA4* (rs3087243), *KIR2DS3*, and *LILRA2* (rs2241524) (Trivioli et al., 2022).

AKT, also known as protein kinase B (PKB), is a serine/threonine kinase and a central regulator of the PI3K/AKT/mTOR pathway, controlling cell survival, metabolism, proliferation, and autophagy (Manning & Toker, 2017). By inhibiting TSC2, a negative regulator of mTORC1, AKT sustains mTORC1 activation (Dibble & Cantley, 2015; Inoki et al., 2002). Consequently, mTORC1 phosphorylates ULK1 at Ser757, preventing its interaction with AMPK and suppressing autophagy initiation (Kim et al., 2011). Additionally, AKT directly phosphorylates ULK1 at Ser774, impairing its autophagy-inducing function, and phosphorylates Beclin-1 at Ser295, disrupting its association with VPS34, which is essential for autophagosome formation (Wang et al., 2012). Furthermore, AKT phosphorylates FoxO1 and FoxO3a, promoting their degradation. Since FoxO transcription factors regulate autophagy-related genes (*e.g.*, LC3, Atg5, Atg7), their inhibition further suppresses autophagy (Schmitt-Ney, 2020). AKT-driven autophagy inhibition is implicated in autoimmune diseases such as rheumatoid arthritis (Keller, Adamopoulos & Lünemann, 2023), systemic lupus erythematosus (Keller, Adamopoulos & Lünemann, 2023; Qi, Zhou & Zhang, 2019), and inflammatory bowel disease (Foerster et al., 2022), exacerbating immune dysregulation and tissue damage. In addition, AKT plays a pivotal role in inflammation by activating the NF-κB pathway, which induces the production of pro-inflammatory cytokines such as TNF-α, IL-6, and IL-1β (Cadwell, 2016; Deretic & Levine, 2018; Matsuzawa-Ishimoto, Hwang & Cadwell, 2018). It influences macrophage polarization, promoting the inflammatory M1 phenotype while suppressing the anti-inflammatory M2 phenotype (Linton, Moslehi & Babaev, 2019; Matsuzawa-Ishimoto, Hwang & Cadwell, 2018). In adaptive immunity, AKT enhances the survival and activation of autoreactive T and B cells, contributing to autoimmune disorders (Cadwell, 2016; Deretic, 2021; Matsuzawa-Ishimoto, Hwang & Cadwell, 2018). Dysregulated AKT signaling disrupts immune balance, leading to persistent inflammation and exacerbating tissue damage in autoimmune diseases (Cadwell, 2016).

ANCA play a critical role in driving inflammation in AAV, a group of autoimmune disorders marked by excessive neutrophil activation and vascular injury (Walulik et al., 2023). Through induction of neutrophil degranulation and reactive oxygen species (ROS) production, ANCA amplify inflammatory cytokine release and endothelial damage (Nakazawa et al., 2019). Autophagy serves a dual function in AAV, balancing neutrophil survival and immune regulation. Chen et al. (2010) identified AKT1 and AKT2—two homologous but distinct genes—as the predominant AKT family members expressed in neutrophils. Given the geographic and gender variations in AAV clinical subtypes and ANCA specificity, we selected four SNPs in AKT1 and three in AKT2 from the 1000 Genomes Project based on their divergent allele frequencies across populations. This selection enabled investigation of the relationship between AKT gene variations and MPA risk, followed by subgroup analyses stratified by P-ANCA status and gender.

## Methods

### Participants

This study included 798 adults, comprising 202 MPA patients and 209 healthy controls recruited from the Second Affiliated Hospital of Guangxi Medical University (September 2009–October 2023), along with 387 Chinese controls from the 1000 Genomes Project (https://www.internationalgenome.org). To enhance statistical power, the two control groups were pooled after verification of genetic homogeneity *via* Chi-square tests in SPSS and logistic regression analysis in SNPStats, which confirmed no significant differences in genotype distributions after accounting for sex (Tables S1–S3). The inclusion criteria for MPA patients were: (a) Diagnosis of MPA based on the 2012 Chapel Hill Consensus Conference on Vasculitis criteria. (b) No history of malignancy, infection, or medication-induced vasculitis. Participant demographic features are summarized in Table 1. The MPA group comprised 76 males and 126 females (mean age: 55.19 ± 14.50 years), and 68.81% (139/202) were P-ANCA positive. The control group consisted of 266 males and 330 females.

**Table 1:** Demographic features of participants.

Characteristic	MPA group (*n* = 202)	Control group (*n* = 596)	
		Guangxi (*n* = 209)	1000 Genomes (*n* = 387)
Gender (M/F)	76/126	82/127	184/203
Age (years, mean ± SD)	55.19 ± 14.50	50.93 ± 13.07	–
P-ANCA (+)	139(68.81%)	–	–

**Notes.**

202 MPA patients and 209 healthy individuals were recruited from the Second Affiliated Hospital of Guangxi Medical University ranging from September 2009 and October 2023. The other 387 Chinese of the control group were from 1000Genomes (https://www.internationalgenome.org).

#### Ethical approval

The study protocol was approved by the Ethics Committee of the Second Affiliated Hospital of Guangxi Medical University (NO. 2018 KY-0100 and 2024 KY-0782) and conducted in strict accordance with the ethical principles outlined in the Declaration of Helsinki. Written informed consent was obtained from all participants prior to their inclusion in the study.

### Methods of obtaining genotypes

Peripheral blood samples were collected from 411 hospital-recruited participants, and DNA was extracted using a Blood DNA Extraction Kit (Tiangen, Beijing, CA). DNA quality was assessed with a Nanodrop 2000 spectrophotometer (Thermo Fisher Scientific, USA), ensuring A260/280 ratios between 1.8–2.0. PCR primers for *AKT1* and *AKT2* were designed and synthesized (Sangon Biotech, Shanghai, CA). The PCR-amplified DNA was purified using AMPure XP beads, followed by quality assessment *via* agarose gel electrophoresis. Whole-genome sequencing was conducted using HiSeq XTen (Illumina, USA). The sequencing reads were processed using Cutadapt (v1.2.1) and PRINSEQ-lite (v0.20.3) to remove adapter sequences and filter low-quality reads. SNP genotyping was performed through BWA (v0.7.13-r1126) for alignment and Samtools (v0.1.18) for variant calling. Additionally, SNP genotype data from 387 individuals were obtained from the 1000 Genomes Project (https://www.internationalgenome.org), serving as a reference population.

### Gene-level associations analysis

Gene-level associations were assessed through two complementary strategies. First, a likelihood-ratio test was implemented in R by comparing logistic regression models with and without the four AKT1 SNPs, while adjusting for AKT2. Second, a set-based association test was performed using PLINK (v1.9), in which the four AKT1 SNPs and three AKT2 SNPs were grouped into separate gene sets. We further conducted exploratory analyses—including linkage disequilibrium estimation, haplotype reconstruction, and SNP-SNP interaction testing—to examine underlying genetic relationships. Finally, tissue-specific cis-eQTL information for relevant SNPs was retrieved from the 3DSNP database (v2.0).

### Statistical analysis

Data statistics were performed *via* SPSS (version 25.0) and SNPStats (https://www.snpstats.net/start.htm). OR value was calculated by logistic regression analysis. Linkage disequilibrium and Haplotype analysis were performed *via* SNPStats and Haploview (version 4.1). Interaction analysis was tested by Generalized multifactor dimensionality reduction (GMDR) (version 0.9). To control the false discovery rate (FDR) in multiple comparisons, we used the Benjamini–Hochberg procedure (BH). Statistical significance was determined by *P*-value <*Q*-value adjusted by FDR (BH).

## Results

### Information of SNPs

**Figure 1:**
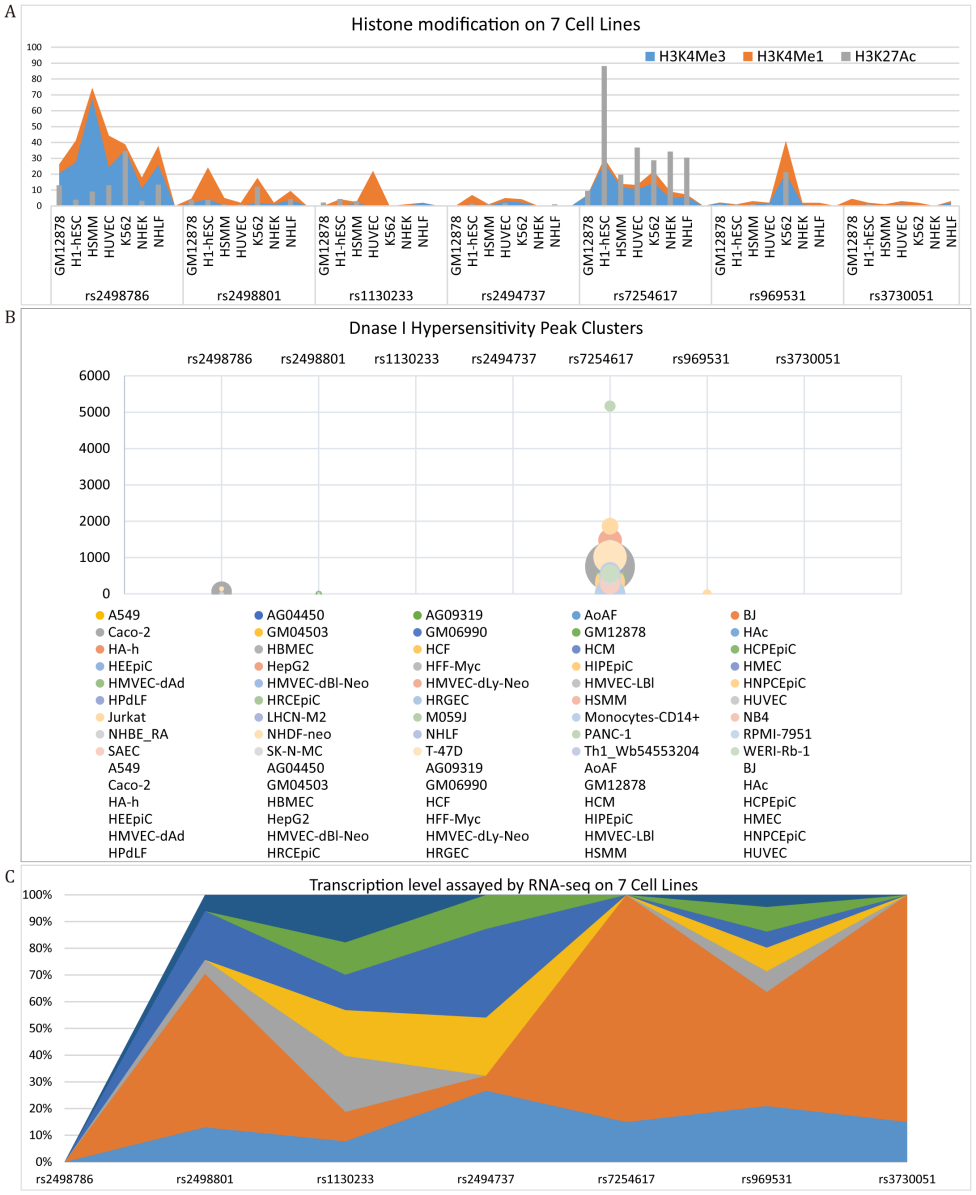
Annotation of SNPs from UCSC (ENCODE). The annotation information for the SNPs includes histone modifications (H3K4me3, H3K4me1, H3K27Ac) (A), DNase I hypersensitivity peak clusters across different cell lines (B) and transcription levels in seven cell lines (C).

In this study, we analyzed seven SNPs from the 1000 Genomes Project, whose allele frequencies exhibited significant variations across different populations (Fig. S1). These included four SNPs in *AKT1* (rs2498786, rs2498801, rs1130233, rs2494737) and three SNPs in *AKT2* (rs7254617, rs969531, rs3730051). A comprehensive characterization of the SNPs was conducted, detailing their genomic locations, predicted functional consequences, and allele frequencies (Table 2, Table S4). This genetic profile was complemented by functional genomic analyses confirming the SNPs’ presence in active regulatory regions (Fig. 1). The rs2498786 and rs7254617 are promoter variants linked to histone modifications (H3K4me3, H3K4me1, H3K27Ac; A) and DNase I hypersensitivity peaks (B) (ENCODE). The rs2498801 and rs969531 function as enhancers. And the rs1130233 (exon) has high transcriptional activity (C). In contrast, the intronic SNPs rs2494737 and rs3730051 were characterized by low levels of associated histone modifications and chromatin accessibility in the ENCODE datasets. The genotypic data for all 798 participants and their association with MPA risk are summarized in Table 2. In this study, the minor allele frequency (MAF) exceeded 0.05 in both the MPA and control groups, thereby satisfying the MAF threshold (MAF >  0.05). Additionally, the Hardy-Weinberg equilibrium (HWE) was maintained (HWE *p* > 0.05) in the control group. Statistically significant associations with MPA risk were identified for specific alleles of AKT1, namely rs2498786 (*P* = 0.003) and rs1130233 (*P* = 0.003). Genotype-based analysis further revealed significant associations for rs2498786 (*P* = 0.015) and rs1130233 (*P* = 0.013). In contrast, neither the alleles nor the genotypes of AKT1 rs2494737 or any of the AKT2 SNPs (rs7254617, rs969531, and rs3730051) showed any association with MPA susceptibility (all *p* > 0.05). Importantly, after FDR-BH adjustment, the initial associations observed for rs2498801 were no longer statistically significant.

**Table 2:** Information of SNPs in AKT1 and AKT2 genes and MPA risk (MPA, *n* = 202; Control, *n* = 596).

SNP	Gene	Alleles	Role	MAF		HWE-p	Allele-p^a^	Genotype-p^a^
				MPA	Control				
rs2498786	AKT1	C>G	Promoter	0.149	0.229	0.628	0.003^**^	0.015^*^
rs2498801		C>T	Enhancer	0.280	0.337	0.410	0.077	0.163
rs1130233		T>C	Exon	0.295	0.393	0.872	0.003^**^	0.013^**^
rs2494737		A>T	Intron	0.285	0.336	0.797	0.096	0.277
rs7254617	AKT2	G>A	Promoter	0.121	0.122	0.084	0.985	1.037
rs969531		C>T	Enhancer	0.255	0.247	0.620	0.923	1.037	
rs3730051		T>C	Intron	0.307	0.295	0.737	0.923	0.752

**Notes.**

Analysis was performed using logistic regression analysis (https://www.snpstats.net/start.htm). *P* value was adjusted by FDR using the Benjamini–Hochberg procedure. Bolded *p*-values indicate statistical significance.

Abbreviations Chr chromosome MAF Minor Allele Frequency MPA MPA Group Control Control Group HWE Hardy–Weinberg equilibrium

p^a^, *P*-value adjusted by FDR (BH).

* *P*-adjusted < 0.05.

** *P*-adjusted < 0.01.

### MPA risk analysis

Association analysis of *AKT1* SNPs with MPA risk was performed under four genetic models (Codominant, Dominant, Recessive and Overdominant) using SNPStats (Table 3). The rs2498786 G and rs1130233 C alleles exhibited a negative association with MPA risk in the Codominant, Dominant, and Recessive models. However, after FDR-BH correction, the association of rs2498801 under the Recessive model was no longer statistically significant. By contrast, neither *AKT1* rs2494737 nor any of the *AKT2* SNPs were associated with MPA risk in any model (Table S5).

**Table 3:** Association between the SNPs in AKT1 and MPA risk (*n* = 798, adjusted by sex).

Loci	Model	Genotype	MPA (*n* = 202)	Control (*n* = 596)	OR (95% CI)	P^a^
rs2498786	Codominant	C/C	145 (71.8%)	356 (59.8%)	1.00	–
		C/G	54 (26.7%)	205 (34.5%)	**0.64 (0.45–0.92)**	0.016^*^
		G/G	3 (1.5%)	34 (5.7%)	**0.22 (0.07–0.71)**	
	Dominant	C/C	145 (71.8%)	356 (59.8%)	1.00	**–**
		C/G-G/G	57 (28.2%)	239 (40.2%)	**0.58 (0.41–0.82)**	0.016^*^
	Recessive	C/C-C/G	199 (98.5%)	561 (94.3%)	1.00	**–**
		G/G	3 (1.5%)	34 (5.7%)	**0.25 (0.08–0.82)**	0.019^*^
	Overdominant	C/C-G/G	148 (73.3%)	390 (65.5%)	1.00	**–**
		C/G	54 (26.7%)	205 (34.5%)	**0.69 (0.48–0.98)**	0.076
rs2498801	Codominant	C/C	101 (50%)	260 (43.7%)	1.00	**–**
		T/C	89 (44.1%)	269 (45.2%)	0.88 (0.63–1.24)	0.116
		T/T	12 (5.9%)	66 (11.1%)	**0.48 (0.25–0.92)**	
	Dominant	C/C	101 (50%)	260 (43.7%)	1.00	–
		T/C-T/T	101 (50%)	335 (56.3%)	0.80 (0.58–1.11)	0.262
	Recessive	C/C-T/C	190 (94.1%)	529 (88.9%)	1.00	**–**
		T/T	12 (5.9%)	66 (11.1%)	**0.51 (0.27–0.96)**	0.059
	Overdominant	C/C-T/T	113 (55.9%)	326 (54.8%)	1.00	**–**
		T/C	89 (44.1%)	269 (45.2%)	0.99 (0.72–1.37)	0.950
rs1130233	Codominant	T/T	97 (48%)	223 (37.5%)	1.00	–
		C/T	91 (45%)	277 (46.5%)	0.77 (0.55–1.08)	0.016^*^
		C/C	14 (6.9%)	95 (16%)	**0.35 (0.19–0.64)**	
	Dominant	T/T	97 (48%)	223 (37.5%)	1.00	**–**
		C/T-C/C	105 (52%)	372 (62.5%)	**0.66 (0.48–0.92)**	0.035^*^
	Recessive	T/T-C/T	188 (93.1%)	500 (84%)	1.00	–
		C/C	14 (6.9%)	95 (16%)	**0.40 (0.22–0.71)**	0.016^*^
	Overdominant	T/T-C/C	111 (55%)	318 (53.5%)	1.00	–
		C/T	91 (45%)	277 (46.5%)	0.96 (0.69–1.32)	0.843
rs2494737	Codominant	A/A	262 (44%)	104 (51.5%)	1.00	**–**
		T/A	265 (44.5%)	81 (40.1%)	0.78 (0.56–1.09)	0.262
		T/T	68 (11.4%)	17 (8.4%)	0.65 (0.36–1.15)	
	Dominant	A/A	262 (44%)	104 (51.5%)	1.00	**–**
		T/A-T/T	333 (56%)	98 (48.5%)	0.75 (0.55–1.04)	0.134
	Recessive	A/A-T/A	527 (88.6%)	185 (91.6%)	1.00	**–**
		T/T	68 (11.4%)	17 (8.4%)	0.73 (0.42–1.27)	0.307
	Overdominant	A/A-T/T	330 (55.5%)	121 (59.9%)	1.00	–
		T/A	265 (44.5%)	81 (40.1%)	0.84 (0.61–1.16)	0.343

**Notes.**

Analysis was performed by SNPStats (https://www.snpstats.net/start.htm). *P* value was adjusted by FDR using the Benjamini–Hochberg procedure. Bolded *p*-values indicate statistical significance.

Abbreviations MPA MPA Group Control Control Group

p^a^, *P*-value adjusted by FDR (BH).

* *P*-adjusted < 0.05.

Subgroup analysis of MPA patients with serum P-ANCA positivity is summarized in Table 4. The rs2498786 polymorphism showed consistent associations with P-ANCA-positive status under Codominant, Dominant, and Recessive models (*P* = 0.024, *P* = 0.024, and *P* = 0.032, respectively), aligning with its association with overall MPA risk. The rs1130233 C allele was negatively associated with P-ANCA positivity in Codominant and Recessive models (*P* = 0.042 and *P* = 0.032). No significant associations were observed for the remaining SNPs in any genetic model (Table S6), consistent with their lack of association in the primary analysis.

**Table 4:** SNPs in *AKT1* and MPA patients with blood P-ANCA (+) susceptibility analysis (*n* = 737, adjusted by sex).

SNP	Model	Genotype	ANCA (*n* = 139)	Control (*n* = 596)	OR (95% CI)	P^a^
rs2498786	Codominant	C/C	101 (72.7%)	356 (59.8%)	1.00	–
		C/G	36 (25.9%)	205 (34.5%)	**0.61 (0.40–0.92)**	**0.024** ^*^
		G/G	2 (1.4%)	34 (5.7%)	**0.20 (0.05–0.86)**	
	Dominant	C/C	101 (72.7%)	356 (59.8%)	1.00	–
		C/G-G/G	38 (27.3%)	239 (40.2%)	**0.55 (0.37–0.83)**	0.024^*^
	Recessive	C/C-C/G	137 (98.6%)	561 (94.3%)	1.00	–
		G/G	2 (1.4%)	34 (5.7%)	0.24 (0.06–1.01)	0.032^*^
	Overdominant	C/C-G/G	103 (74.1%)	390 (65.5%)	1.00	–
		C/G	36 (25.9%)	205 (34.5%)	**0.65 (0.43–0.99)**	0.055
rs1130233	Codominant	T/T	64 (46%)	223 (37.5%)	1.00	–
		C/T	64 (46%)	277 (46.5%)	0.82 (0.56–1.22)	0.042^*^
		C/C	11 (7.9%)	95 (16%)	**0.41 (0.21–0.82)**	
	Dominant	T/T	64 (46%)	223 (37.5%)	1.00	–
		C/T-C/C	75 (54%)	372 (62.5%)	0.72 (0.50–1.05)	0.098
	Recessive	T/T-C/T	128 (92.1%)	500 (84%)	1.00	–
		C/C	11 (7.9%)	95 (16%)	**0.46 (0.24–0.88)**	0.032^*^
	Overdominant	T/T-C/C	75 (54%)	318 (53.5%)	1.00	–
		C/T	64 (46%)	277 (46.5%)	1.00 (0.69–1.44)	0.980

**Notes.**

Analysis was performed by SNPStats (https://www.snpstats.net/start.htm). *P* value was adjusted by FDR using the Benjamini–Hochberg procedure. Bolded *p*-values indicate statistical significance.

Abbreviations ANCA MPA patients with blood P-ANCA (+) Group. Control Control Group

P^a^, *P*-value adjusted by FDR (BH).

* *P*-adjusted < 0.05.

A significant interaction with gender was observed for the rs2498786 genotype (*P*-interaction = 0.007; Table 5), whereas no such interaction was identified for any other tested SNPs. This analysis was conducted *via* logistic regression on the SNPStats platform, with adjustments for the relevant SNP and gender.

**Table 5:** Interaction analysis between SNPs in AKT1 (rs2498786) and gender (*n* = 796).

Loci	Female			Male			Interaction *P*-value
	MPA	Control	OR (95% CI)	MPA	Control	OR (95% CI)	
	98	170	1.0	47	186	0.52 (0.35-0.79)	
rs2498786	26	80	0.39 (0.24–0.64)	28	125	0.66 (0.40–1.09)	0.007^**^
	2	15	0.20 (0.05–0.88)	1	19	0.13 (0.02–0.97)	

**Notes.**

Analysis was performed by SNPStats (https://www.snpstats.net/start.htm).

Abbreviations MPA MPA Group Control Control Group

** *P* < 0.01.

### Gene-level evidence from multivariable logistic regression and set-based analysis

A gene-level analysis was conducted to assess the combined contribution of AKT1 polymorphisms. Using a likelihood-ratio test that compared a full model incorporating all AKT1 and AKT2 SNPs against a reduced model containing only AKT2 variants, we found the AKT1 gene block to be independently associated with MPA susceptibility (*χ*^2^ = 24.885, *df* = 4, *P* = 5.31 × 10^−5^), supporting its role as a risk locus (Table S7). Then, gene-set analyses were performed using PLINK to examine the aggregated effects of AKT1 and AKT2 variants. The analysis identified a significant association between the AKT1 gene set (4 SNPs) and decreased MPA risk (OR = 0.884, 95% CI [0.820–0.953]; *P* = 0.002), which persisted after FDR adjustment (*P*-adjusted = 0.004). Conversely, the AKT2 gene set (3 SNPs) demonstrated no significant relationship with disease susceptibility (OR = 1.028, 95% CI [0.902–1.171]; *P* = 0.686; *P*-adjusted = 0.686) (Table S8). These findings collectively indicate a protective function specifically associated with AKT1, with no parallel effect observed for AKT2 in MPA development.

### Exploratory analysis of SNP-SNP interactions

To evaluate potential genetic relationships, we performed a triad of genetic analyses: linkage disequilibrium assessment, haplotype construction, and SNP-SNP interaction testing.

#### Linkage disequilibrium analysis

**Figure 2:**
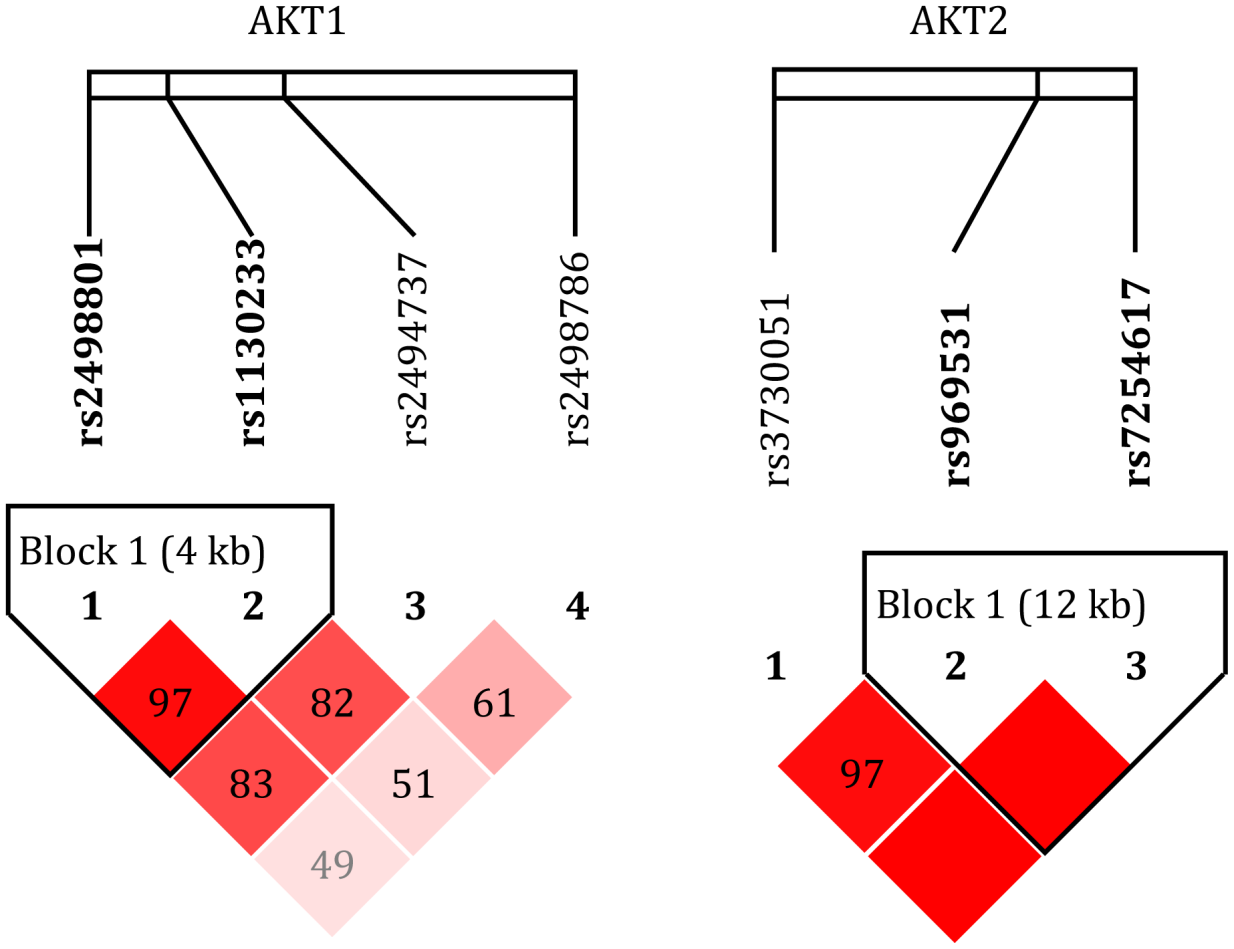
Linkage disequilibrium plot. The linkage disequilibrium plot of the SNPs (rs2498786, rs2498801, rs1130233, rs2494737, rs7254617, rs969531, and rs3730051) was generated using Haploview (version 4.1). The intensity of the color corresponds to the degree of linkage disequilibrium, with darker colors indicating values closer to 100, representing a higher likelihood of linkage disequilibrium. The AKT1 and AKT2 loci exhibited linkage imbalance independently.

Analysis revealed no significant linkage disequilibrium between SNPs located in the AKT1 and AKT2 gene. However, strong linkage was observed within each gene: the four AKT1 SNPs (rs2498786, rs2498801, rs1130233, and rs2494737) and the three AKT2 SNPs (rs7254617, rs969531, and rs3730051) constituted independent linkage blocks. Specific D’ measurement values are annotated in the corresponding boxes in Fig. 2.

#### Haplotype analysis

Haplotype analysis using the SNPStats platform identified two AKT1 haplotypes significantly associated with MPA risk. Haplotypes G-T-C-T (*P*-adjusted = 0.003) and C-C-C-A (*P*-adjusted = 0.025) both demonstrated clear protective effects (Table 6). This indicates that individuals carrying these specific haplotype combinations have a significantly reduced risk of developing MPA.

**Table 6:** Haplotypes analysis of AKT1 (rs2498786/rs2498801/rs1130233/rs2494737) and MPA risk (adjusted by sex).

AKT1 SNP	Haplotypes	MPA (%)	Control (%)	OR (95%CI)	*P*-adjusted
rs2498786/ rs2498801/ rs1130233/ rs2494737	C-C-T-A	62.16	53.11	1.00	–
	C-T-C-T	15.54	14.69	0.96 (0.67–1.37)	1.134
	G-T-C-T	8.33	14.93	**0.46 (0.30–0.71)**	0.003^**^
	G-C-T-A	4.80	3.99	0.99 (0.53–1.86)	1.134
	C-C-C-A	1.51	4.40	**0.29 (0.12–0.71)**	0.025^*^
	C-T-C-A	2.98	3.23	0.78 (0.38–1.62)	0.893
	G-C-T-T	1.64	2.34	0.58 (0.23–1.44)	0.560
	rare	3.04	3.31	0.92 (0.45–1.86)	1.134

**Notes.**

Global haplotype association *p*-value: 0.001. rare, Haplotypes other than those listed above. Analysis was performed by SNPStats (https://www.snpstats.net/start.htm). *P* value was adjusted by FDR using the Benjamini–Hochberg procedure. Bolded *p*-values indicate statistical significance.

Abbreviations MPA MPA group Control Control group

* *P*-adjusted < 0.05.

** *P*-adjusted < 0.01.

#### Interaction analysis

**Figure 3:**
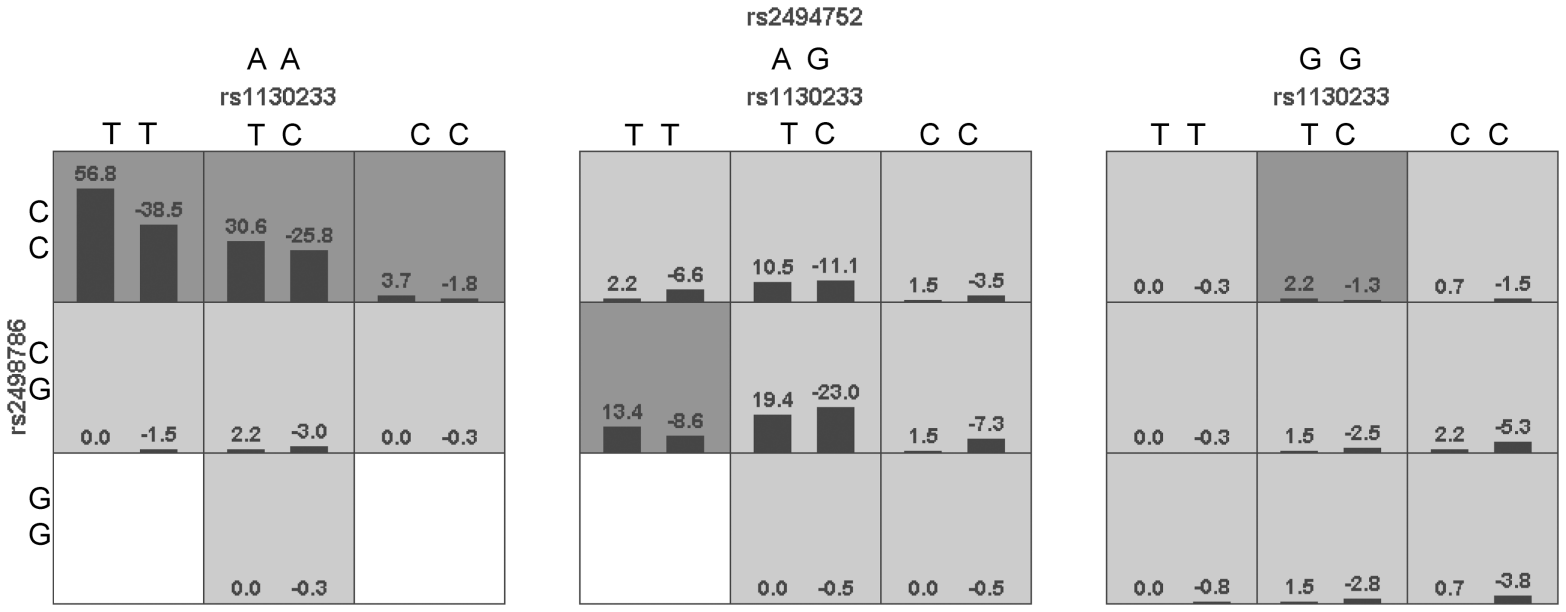
Interactions among SNPs related to MPA risk in the best model. The interactions among SNPs in the model (rs2498786/rs2494752/rs1130233) were visualized using generalized multifactor dimensionality reduction (GMDR, version 4.1). The left bars in each cell represent case positive scores, while the right bars indicate control negative scores. Higher positive scores correspond to an increased risk of MPA. The genotype combination rs2498786 CC, rs2494752 AA, and rs1130233 TT was associated with the highest risk of MPA (*P* = 0.001).

Using the Generalized Multifactor Dimensionality Reduction (GMDR) method, we analyzed interactions among SNPs screened in this study and previous research (Li et al., 2023). Among various models, the combination of rs2498786, rs2494752, and rs1130233 performed most prominently, achieving a perfect cross-validation consistency score (10/10) and a testing balanced accuracy of 0.5805. The risk analysis schematic (Fig. 3) visually demonstrates that the genotype combination rs2498786 CC, rs2494752 AA, and rs1130233 TT elevates the risk of MPA to the highest level. Complete analysis data are provided in Table S9.

### eQTL analysis of MPA-associated *AKT1* variants

**Figure 4:**
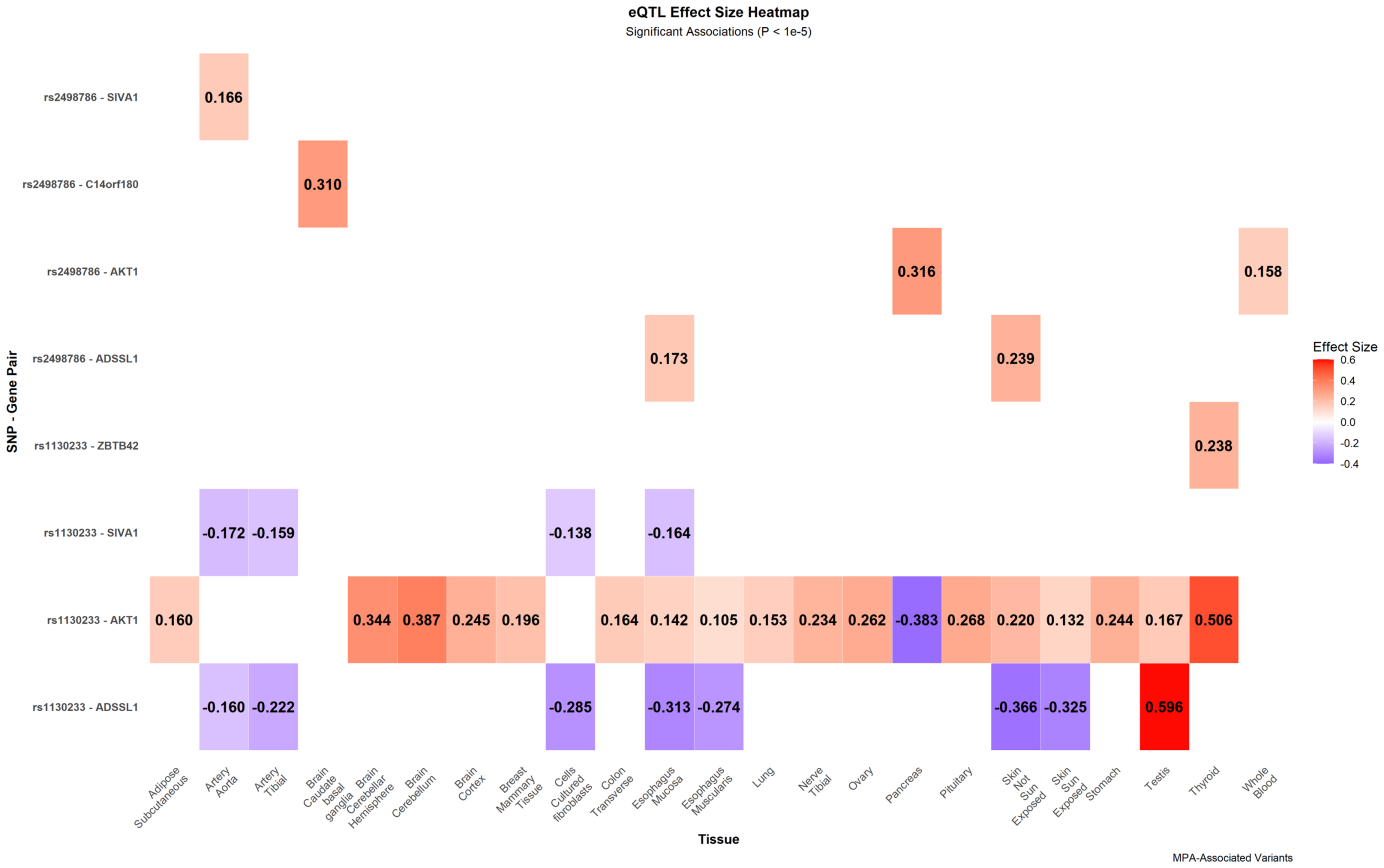
Tissue-specific eQTL landscape of AKT1 variants associated with MPA. Effect sizes of significant *cis*-eQTL associations for MPA-linked *AKT1* SNPs (rs2498786 and rs1130233) across human tissues (3DSNP v2.0), with color intensity representing the direction of allele effect on expression (red: positive; blue: negative). eQTL, expression quantitative trait locus; MPA, microscopic polyangiitis.

Expression quantitative trait locus (eQTL) analysis revealed that the MPA-protective alleles of AKT1 SNPs function as significant cis-eQTLs (Fig. 4). The rs2498786-G allele was associated with enhanced AKT1 expression in whole blood (*P* = 2.99  × 10^−10^) and pancreas (*P* = 8.96  × 10^−8^). Similarly, the rs1130233-C allele significantly upregulated AKT1 expression across multiple tissues, most notably in thyroid (*P* = 1.13  × 10^−40^) and whole blood. Both variants additionally influenced the expression of nearby genes, including SIVA1, in vascular and immune-relevant tissues. These findings indicate that the protective association of these SNPs likely operates through the tissue-specific transcriptional regulation of AKT1 and its regulatory network.

## Discussion

This study presents accumulating evidence suggesting that the *AKT1* gene, in contrast to its homologous counterpart *AKT2*, may contribute to MPA susceptibility. Our multi-level analyses are consistent with a model in which specific *AKT1* variants could confer protection through mechanisms relevant to AAV pathology, whereas *AKT2* appears to lack a comparable association.

The functional characteristics of the investigated SNPs provide insights into their potential regulatory roles. The promoter locus rs2498786 reside within genomic regions marked by characteristic histone modifications and DNase I hypersensitivity, indicating their potential as regulatory elements (Lawrence, Daujat & Schneider, 2016; Field & Adelman, 2020). Notably, rs2498786 has been previously associated with altered AKT1 protein levels (Liu et al., 2015). The exonic variant rs1130233, characterized by high transcriptional activity, may influence mRNA stability or protein function (He et al., 2023), a notion further supported by observations of genotype-dependent protein levels (Zubair, Khan & Imran, 2022).

The protective associations of promoter variant rs2498786 and exonic variant rs1130233 with MPA risk, particularly their correlation with P-ANCA positivity—a key diagnostic marker observed in 68.81% of our cohort and 55–70% of historical MPA cases (Hunter et al., 2020; Walulik et al., 2023)—lend support to AKT1’s potential role in AAV-related immune dysregulation. The observed association between protective alleles of these SNPs and enhanced AKT1 expression in whole blood suggests a plausible biological mechanism. Within the context of AAV pathogenesis, where ANCA-mediated Fc-Fc*γ* receptor engagement promotes neutrophil activation and NETosis *via* NADPH oxidase-derived ROS (Lu et al., 2021; Shiratori-Aso & Nakazawa, 2023), AKT1’s established function in neutrophil regulatory processes (Chen et al., 2010) raises the possibility that these protective variants may moderate neutrophil hyperactivation and NET formation. This could potentially disrupt the self-perpetuating “ANCA-NETs-ANCA” cycle characteristic of severe vasculitis (Nakazawa et al., 2019), suggesting a potential mechanism linking genetic variation to disease pathology.

Beyond innate immunity, the observed gender interaction with rs2498786, combined with AKT1’s fundamental roles in T and B cell survival and activation (Di Lorenzo et al., 2009; Nagai, Kurebayashi & Koyasu, 2013), extends its potential influence to adaptive immune dysregulation in AAV. The allele-dependent eQTL signals in vascular tissues (aorta, tibial artery) indicate an additional endothelial protective mechanism under ANCA-mediated stress, which aligns with the recognized importance of endothelial integrity in AAV pathogenesis (Dömer et al., 2021; Massicotte-Azarniouch et al., 2022). The coordinated regulation of SIVA1, a gene involved in apoptosis and immune signaling (Shamshul & Yoshio, 2020; Coccia, Solé & Comella, 2021), further supports AKT1’s position within a broader regulatory network maintaining vascular integrity during inflammatory challenge (Lucotte et al., 2023).

Our gene-level analyses, though based on a set of seven SNPs, provide preliminary yet important evidence for the specific involvement of AKT1. The significant association of the AKT1 gene set with reduced MPA risk, contrasted with the null finding for the AKT2 set, underscores the biological specificity of this relationship. This divergence aligns with the well-documented functional differences between these homologous genes: AKT1 drives fundamental immune processes and is highly expressed in immune and vascular tissues (Manning & Toker, 2017), whereas AKT2 exhibits tissue-specific expression with greater emphasis on metabolic regulation and primarily participates in cellular metabolism rather than immune regulation within immune cells (Cho et al., 2001; Kohn et al., 1996). This fundamental distinction, evident in their roles in macrophage polarization (Arranz et al., 2012; Vergadi et al., 2017) and neutrophil regulation (Chen et al., 2010), may explain why AKT1 variants influence MPA susceptibility while AKT2 polymorphisms do not, suggesting our findings reflect specific biological mechanisms rather than general immune pathway disturbances (Chou et al., 2022; Hers, Vincent & Tavaré, 2011).

The haplotype and interaction analyses further substantiate AKT1’s central role in MPA susceptibility. The identification of protective haplotypes (G-T-C-T, C-C-C-A) and the high-risk genotype combination (rs2498786 CC, rs2494752 AA, rs1130233 TT) demonstrates that MPA risk is shaped by complex genetic interactions beyond individual variant effects. These epistatic relationships reveal the intricate genetic architecture underlying this autoimmune vasculitis and highlight the potential utility of multi-locus genetic profiles in risk prediction.

In conclusion, our integrated genetic analysis positions *AKT1* as a significant genetic determinant of MPA susceptibility through mechanisms specifically relevant to AAV pathology. The protective effects appear to operate through modulation of neutrophil activation, regulation of adaptive immunity, and protection of vascular integrity—all central pathways in AAV pathogenesis. The consistent absence of association with *AKT2* variants across all analytical approaches reinforces the specificity of this relationship. These findings not only advance our understanding of MPA genetics but also suggest that targeted modulation of AKT1 signaling may represent a promising therapeutic strategy for this autoimmune vasculitis.

## Conclusions

In summary, this work contributes to growing evidence that AKT1, unlike AKT2, may play a role in MPA susceptibility. The protective associations observed may be mediated through pathways central to ANCA-associated vasculitis, such as those governing neutrophil behavior, immune regulation, and vascular function. These genetic observations help refine the MPA susceptibility landscape and could inform future exploration of AKT1-targeted therapeutic avenues. Further investigation in independent populations and functional characterization of these variants would strengthen these preliminary findings.

## Supplemental Information

10.7717/peerj.20791/supp-1 Supplemental Information 1 Association analysis of AKT1 and AKT2 SNPs between the two control groups under different genetic models (*N* = 597, adjusted by sex) The two control groups were pooled after verification of genetic homogeneity *via* logistic regression analysis in SNPStats.

10.7717/peerj.20791/supp-2 Supplemental Information 2 Chi-square test analysis of genotype distributions between control groups The two control groups were pooled after verification of genetic homogeneity *via* Chi-square tests in SPSS.

10.7717/peerj.20791/supp-3 Supplemental Information 3 Chi-square test analysis of genotype distributions between control groups by gender The two control groups were pooled after verification of genetic homogeneity *via* Chi-square tests in SPSS.

10.7717/peerj.20791/supp-4 Supplemental Information 4 Information of SNPs in AKT1 and AKT2 A comprehensive characterization of the SNPs include their genomic locations, predicted functional consequences

10.7717/peerj.20791/supp-5 Supplemental Information 5 Association between the AKT2 genotypes and MPA risk (*n* = 798, adjusted by sex) Association between the AKT2 genotypes and MPA risk

10.7717/peerj.20791/supp-6 Supplemental Information 6 SNPs ( rs2498801, rs2494737, rs7254617, rs969531 and rs3730051) and MPA patients with blood P_ANCA (+) susceptibility analysis (*n* = 737, adjusted by sex) No significant associations were observed for the remaining SNPs ( rs2498801, rs2494737, rs7254617, rs969531 and rs3730051) in any genetic model in blood P_ANCA (+) susceptibility analysis

10.7717/peerj.20791/supp-7 Supplemental Information 7 Assessing the Collective Association of the AKT1 Gene Block with MPA Risk adjusted by sex the AKT1 gene block to be independently associated with MPA susceptibility

10.7717/peerj.20791/supp-8 Supplemental Information 8 Association of AKT1 and AKT2 Gene Sets with MPA Risk adjusted by sex A significant association between the AKT1 gene set (4 SNPs) and decreased MPA risk.

10.7717/peerj.20791/supp-9 Supplemental Information 9 SNP-SNP interaction analysis performed by GMDR

10.7717/peerj.20791/supp-10 Supplemental Information 10 Annotion of SNPs Data of Figs. 1 and 4

10.7717/peerj.20791/supp-11 Supplemental Information 11 Distribution of alleles among populations The allelic frequency distribution across major populations (East Asian, African, Amerindian, and European) in both the 1000 Genomes Project and this study cohort.Data for the 1000 Genomes populations were sourced from publicly available repositories hosted by the National Center for Biotechnology Information (NCBI; https://www.ncbi.nlm.nih.gov/) and the International Genome Sample Resource (IGSR; https://www.internationalgenome.org/).

10.7717/peerj.20791/supp-12 Supplemental Information 12 Details of VCF file conversions using the IGSR-supported Ensembl tool

## Abbreviations

MPA: Microscopic Polyangiitis
AAV: ANCA-Associated Vasculitis
ANCA: Antineutrophil Cytoplasmic Antibody
SNP: Single Nucleotide Polymorphism
HLA: Human Leukocyte Antigen
PTPN22: Protein Tyrosine Phosphatase Non-Receptor Type 22
FCGR3B: Fc Gamma Receptor IIIb
TLR9: Toll-Like Receptor 9
BACH2: BTB Domain and CNC Homolog 2
CTLA4: Cytotoxic T-Lymphocyte-Associated Protein 4
LILRA2: Leukocyte Immunoglobulin-Like Receptor Subfamily A Member 2
AKT: Protein Kinase B (PKB)
PI3K: Phosphoinositide 3-Kinase
mTOR: Mechanistic Target of Rapamycin
TSC2: Tuberous Sclerosis Complex 2
ULK1: Unc-51 Like Autophagy Activating Kinase 1
AMPK: AMP-Activated Protein Kinase
FoxO: Forkhead Box O
NF-κB: Nuclear Factor Kappa B
TNF-α: Tumor Necrosis Factor Alpha
IL-6: Interleukin 6
IL-1β: Interleukin 1 Beta
Th1: T Helper 1
Th17: T Helper 17
IFN-*γ*: Interferon Gamma
IL-17: Interleukin 17
NETs: Neutrophil Extracellular Traps
NADPH: Nicotinamide Adenine Dinucleotide Phosphate
ROS: Reactive Oxygen Species
Fc-Fcy: Fc Fragment of IgG
PR3: Proteinase 3
MPO: Myeloperoxidase
GMDR: Generalized Multifactor Dimensionality Reduction
HWE: Hardy-Weinberg Equilibrium
MAF: Minor Allele Frequency
PCR: Polymerase Chain Reaction
H3K4me3: Trimethylation of Lysine 4 on Histone H3
H3K4me1: Monomethylation of Lysine 4 on Histone H3
H3K27Ac: Acetylation of Lysine 27 on Histone H3
DNase I: Deoxyribonuclease I
EGFR-TKIs: Epidermal Growth Factor Receptor Tyrosine Kinase Inhibitors
SMAD3: Mothers Against Decapentaplegic Homolog 3
YY1: Yin Yang 1
CD80/86: Cluster of Differentiation 80/86
IL-12: Interleukin 12

## Additional Information and Declarations

### Data Availability

The following information was supplied regarding data availability: The data is available in the Supplemental Files. The Sample Population data is available at the International Genome Sample Resource (IGSR): https://www.internationalgenome.org/data-portal/sample
The 1000 Genomes Project Phase 3 data is available at the International Genome Sample Resource (IGSR): https://www.internationalgenome.org/data-portal/data-collection/phase-3
VCF files were converted to PED/MAP format using the IGSR-supported Ensembl tool: https://grch37.ensembl.org/Multi/Tools/VcftoPed
– Details regarding file conversion can be found in the Supplemental File.

### Clinical Trial Registration

The following information was supplied regarding Clinical Trial registration: 2018 KY-0100 and 2024 KY-0782
