# Supplementary material for: AKT1 but not AKT2 single nucleotide polymorphisms are associated with the risk of microscopic polyangiitis
Source: PeerJ. 2026 Feb 16;14:e20791. doi: 10.7717/peerj.20791 (PMC12919311; doi:10.7717/peerj.20791)
Supplement: Supplemental Information 1 — The two control groups were pooled after verification of genetic homogeneity via logistic regression analysis in SNPStats. [file peerj-14-20791-s001.docx]

**Supplemental Table 1.** Association Analysis of AKT1 and AKT2 SNPs Between the Two Control Groups Under Different Genetic Models (N=597, adjusted by sex)

| SNP | Genetic  Model | Geno-  type | Control-G  N=209 (%) | Control-1000  N=387 (%) | OR (95% CI) | P-value | P-adjusted |
| --- | --- | --- | --- | --- | --- | --- | --- |
| AKT1  rs2498786 | Co-dominant | C/C | 116 (56%) | 226 (61.9%) | 1.00 | 0.140 | 0.327 |
|  |  | C/G | 85 (41.1%) | 114 (31.2%) | 1.54 (0.98-2.42) |  |  |
|  |  | G/G | 6 (2.9%) | 25 (6.8%) | 0.82 (0.28-2.44) |  |  |
|  | Dominant | C/C | 116 (56%) | 226 (61.9%) | 1.00 | 0.110 | 0.305 |
|  |  | C/G-G/G | 91 (44%) | 139 (38.1%) | 1.43 (0.93-2.21) |  |  |
|  | Recessive | C/C-C/G | 201 (97.1%) | 340 (93.2%) | 1.00 | 0.530 | 0.725 |
|  |  | G/G | 6 (2.9%) | 25 (6.8%) | 0.71 (0.24-2.09) |  |  |
|  | Over-dominant | C/C-G/G | 122 (58.9%) | 251 (68.8%) | 1.00 | 0.052 | 0.294 |
|  |  | C/G | 85 (41.1%) | 114 (31.2%) | 1.56 (1.00-2.44) |  |  |
| AKT1  rs2498801 | Co-dominant | C/C | 105 (50.7%) | 146 (40%) | 1.00 | 0.052 | 0.294 |
|  |  | T/C | 85 (41.1%) | 171 (46.9%) | 0.89 (0.58-1.39) |  |  |
|  |  | T/T | 17 (8.2%) | 48 (13.2%) | 0.38 (0.16-0.87) |  |  |
|  | Dominant | C/C | 105 (50.7%) | 146 (40%) | 1.00 | 0.230 | 0.460 |
|  |  | T/C-T/T | 102 (49.3%) | 219 (60%) | 0.77 (0.51-1.18) |  |  |
|  | Recessive | C/C-T/C | 190 (91.8%) | 317 (86.8%) | 1.00 | **0.018** | 0.294 |
|  |  | T/T | 17 (8.2%) | 48 (13.2%) | 0.40 (0.18-0.90) |  |  |
|  | Over-dominant | C/C-T/T | 122 (58.9%) | 194 (53.1%) | 1.00 | 0.850 | 0.881 |
|  |  | T/C | 85 (41.1%) | 171 (46.9%) | 1.04 (0.68-1.59) |  |  |
| AKT1  rs1130233 | Co-dominant | T/T | 98 (47.3%) | 115 (31.5%) | 1.00 | 0.098 | 0.305 |
|  |  | C/T | 88 (42.5%) | 177 (48.5%) | 0.78 (0.50-1.22) |  |  |
|  |  | C/C | 21 (10.1%) | 73 (20%) | 0.44 (0.20-0.97) |  |  |
|  | Dominant | T/T | 98 (47.3%) | 115 (31.5%) | 1.00 | 0.110 | 0.305 |
|  |  | C/T-C/C | 109 (52.7%) | 250 (68.5%) | 0.71 (0.46-1.09) |  |  |
|  | Recessive | T/T-C/T | 186 (89.9%) | 292 (80%) | 1.00 | 0.063 | 0.294 |
|  |  | C/C | 21 (10.1%) | 73 (20%) | 0.50 (0.24-1.07) |  |  |
|  | Over-dominant | T/T-C/C | 119 (57.5%) | 188 (51.5%) | 1.00 | 0.650 | 0.758 |
|  |  | C/T | 88 (42.5%) | 177 (48.5%) | 0.91 (0.60-1.38) |  |  |
| AKT1  rs2494737 | Co-dominant | A/A | 99 (47.8%) | 155 (42.5%) | 1.00 | 0.077 | 0.305 |
|  |  | T/A | 91 (44%) | 164 (44.9%) | 0.99 (0.64-1.54) |  |  |
|  |  | T/T | 17 (8.2%) | 46 (12.6%) | 0.41 (0.18-0.95) |  |  |
|  | Dominant | A/A | 99 (47.8%) | 155 (42.5%) | 1.00 | 0.480 | 0.707 |
|  |  | T/A-T/T | 108 (52.2%) | 210 (57.5%) | 0.86 (0.56-1.31) |  |  |
|  | Recessive | A/A-T/A | 190 (91.8%) | 319 (87.4%) | 1.00 | **0.024** | 0.294 |
|  |  | T/T | 17 (8.2%) | 46 (12.6%) | 0.41 (0.18-0.93) |  |  |
|  | Over-dominant | A/A-T/T | 116 (56%) | 201 (55.1%) | 1.00 | 0.550 | 0.725 |
|  |  | T/A | 91 (44%) | 164 (44.9%) | 1.14 (0.75-1.73) |  |  |
|  | Co-dominant | G/G | 166 (80.2%) | 280 (76.7%) | 1.00 | 0.120 | 0.305 |
|  |  | G/A | 40 (19.3%) | 75 (20.6%) | 0.78 (0.46-1.32) |  |  |
| AKT2  rs7254617 |  | A/A | 1 (0.5%) | 10 (2.7%) | 0.18 (0.02-1.49) |  |  |
|  | Dominant | G/G | 166 (80.2%) | 280 (76.7%) | 1.00 | 0.170 | 0.366 |
|  |  | G/A-A/A | 41 (19.8%) | 85 (23.3%) | 0.70 (0.42-1.17) |  |  |
|  | Recessive | G/G-G/A | 206 (99.5%) | 355 (97.3%) | 1.00 | 0.062 | 0.294 |
|  |  | A/A | 1 (0.5%) | 10 (2.7%) | 0.19 (0.02-1.57) |  |  |
|  | Over-dominant | G/G-A/A | 167 (80.7%) | 290 (79.5%) | 1.00 | 0.440 | 0.707 |
|  |  | G/A | 40 (19.3%) | 75 (20.6%) | 0.81 (0.48-1.37) |  |  |
| AKT2  rs969531 | Co-dominant | T/T | 102 (49.3%) | 186 (51%) | 1.00 | 0.460 | 0.707 |
|  |  | T/C | 84 (40.6%) | 152 (41.6%) | 0.86 (0.55-1.34) |  |  |
|  |  | C/C | 21 (10.1%) | 27 (7.4%) | 1.36 (0.66-2.79) |  |  |
|  | Dominant | T/T | 102 (49.3%) | 186 (51%) | 1.00 | 0.770 | 0.862 |
|  |  | T/C-C/C | 105 (50.7%) | 179 (49%) | 0.94 (0.62-1.43) |  |  |
|  | Recessive | T/T-T/C | 186 (89.9%) | 338 (92.6%) | 1.00 | 0.290 | 0.541 |
|  |  | C/C | 21 (10.1%) | 27 (7.4%) | 1.45 (0.73-2.90) |  |  |
|  | Over-dominant | T/T-C/C | 123 (59.4%) | 213 (58.4%) | 1.00 | 0.350 | 0.613 |
|  |  | T/C | 84 (40.6%) | 152 (41.6%) | 0.82 (0.53-1.25) |  |  |
| AKT2  rs3730051 | Co-dominant | T/T | 214 (58.6%) | 117 (56.5%) | 1.00 | 0.850 | 0.881 |
|  |  | T/C | 131 (35.9%) | 74 (35.8%) | 0.88 (0.57-1.38) |  |  |
|  |  | C/C | 20 (5.5%) | 16 (7.7%) | 0.99 (0.45-2.17) |  |  |
|  | Dominant | T/T | 214 (58.6%) | 117 (56.5%) | 1.00 | 0.630 | 0.758 |
|  |  | T/C-C/C | 151 (41.4%) | 90 (43.5%) | 0.90 (0.59-1.37) |  |  |
|  | Recessive | T/T-T/C | 345 (94.5%) | 191 (92.3%) | 1.00 | 0.910 | 0.910 |
|  |  | C/C | 20 (5.5%) | 16 (7.7%) | 1.04 (0.49-2.24) |  |  |
|  | Over-dominant | T/T-C/C | 234 (64.1%) | 133 (64.2%) | 1.00 | 0.570 | 0.725 |
|  |  | T/C | 131 (35.9%) | 74 (35.8%) | 0.88 (0.57-1.36) |  |  |

**Note:** Genetic homogeneity between the two control groups was assessed using logistic regression analysis implemented in SNPStats, with adjustment for sex. P value was adjusted by FDR using the Benjamini-Hochberg procedure. Bolded p-values indicate statistical significance.

**Abbreviations:** Control_G, healthy adults recruited in the current study; Control_1000, healthy adults from the 1000 Genomes Project; OR, odds ratio; CI, confidence interval.
