# Supplementary material for: AKT1 but not AKT2 single nucleotide polymorphisms are associated with the risk of microscopic polyangiitis
Source: PeerJ. 2026 Feb 16;14:e20791. doi: 10.7717/peerj.20791 (PMC12919311; doi:10.7717/peerj.20791)
Supplement: Supplemental Information 2 — The two control groups were pooled after verification of genetic homogeneity via Chi-square tests in SPSS. [file peerj-14-20791-s002.docx]

**Supplemental Table 2.** Chi-square Test Analysis of Genotype Distributions Between Control Groups

| SNP | Genotype | Control_G (N=209) n (%) | Control_1000 (N=387) n (%) | χ² | df | | P | P-adjusted |
| --- | --- | --- | --- | --- | --- | --- | --- | --- |
| rs1130233 | C/C | 21 (22.1%) | 74 (77.9%) | 16.668 | 2 | | <0.001 | **0.002**** |
|  | C/T | 89 (32.0%) | 189 (68.0%) |  |  |  |  |  |
|  | T/T | 99 (44.4%) | 124 (55.6%) |  |  |  |  |  |
| rs2498801 | C/C | 105 (40.4%) | 155 (59.6%) | 6.700 | 2 | | 0.035 | 0.082 |
|  | T/C | 87 (32.2%) | 183 (67.8%) |  |  |  |  |  |
|  | T/T | 17 (25.8%) | 49 (74.2%) |  |  |  |  |  |
| rs2494737 | A/A | 100 (38.0%) | 163 (62.0%) | 4.050 | 2 | | 0.132 | 0.185 |
|  | T/A | 92 (34.7%) | 173 (65.3%) |  |  |  |  |  |
|  | T/T | 17 (25.0%) | 51 (75.0%) |  |  |  |  |  |
| rs2498786 | C/C | 118 (33.1%) | 239 (66.9%) | 8.850 | 2 | | 0.012 | **0.042*** |
|  | C/G | 85 (41.5%) | 120 (58.5%) |  |  |  |  |  |
|  | G/G | 6 (17.6%) | 28 (82.4%) |  |  |  |  |  |
| rs3730051 | C/C | 16 (43.2%) | 21 (56.8%) | 1.214 | 2 | | 0.545 | 0.572 |
|  | T/C | 75 (33.9%) | 146 (66.1%) |  |  |  |  |  |
|  | T/T | 118 (34.9%) | 220 (65.1%) |  |  |  |  |  |
| rs7254617 | A/A | 1 (7.7%) | 12 (92.3%) | 4.654 | 2 | | 0.098 | 0.171 |
|  | G/A | 40 (33.6%) | 79 (66.4%) |  |  |  |  |  |
|  | G/G | 168 (36.2%) | 296 (63.8%) |  |  |  |  |  |
| rs969531 | C/C | 103 (35.0%) | 191 (65.0%) | 1.253 | 2 | 0.534 | | 0.572 |
|  | T/C | 85 (33.7%) | 167 (66.3%) |  |  |  |  |  |
|  | T/T | 21 (42.0%) | 29 (58.0%) |  |  |  |  |  |

Note: The percentages shown are row percentages, indicating the proportion of each genotype falling into the two control groups. P value was adjusted by FDR using the Benjamini-Hochberg procedure. Bolded p-values indicate statistical significance.

Abbreviations: Control_G, healthy adults recruited in the current study; Control_1000, healthy adults from the 1000 Genomes Project; df, degrees of freedom. *, P-adjusted ＜0.05. **, P-adjusted ＜0.01.
