# Supplementary material for: AKT1 but not AKT2 single nucleotide polymorphisms are associated with the risk of microscopic polyangiitis
Source: PeerJ. 2026 Feb 16;14:e20791. doi: 10.7717/peerj.20791 (PMC12919311; doi:10.7717/peerj.20791)
Supplement: Supplemental Information 3 — The two control groups were pooled after verification of genetic homogeneity via Chi-square tests in SPSS. [file peerj-14-20791-s003.docx]

**Supplemental Table 3.** Chi-square Test Analysis of Genotype Distributions Between Control Groups by gender

| SNP | Gender | χ² | df | P | P-adjusted |
| --- | --- | --- | --- | --- | --- |
| rs1130233 | Female | 5.08 | 2 | 0.079 | 0.095 |
|  | Male | 7.01 | 2 | 0.030 | 0.060 |
|  | Total | 13.82 | 2 | <0.001 | **0.006**** |
| rs2498786 | Female | 6.44 | 2 | 0.040 | 0.060 |
|  | Male | 1.76 | 2 | 0.415 | 0.415 |
|  | Total | 8.850 | 2 | 0.012 | **0.036*** |

Note: P value was adjusted by FDR using the Benjamini-Hochberg procedure. Bolded p-values indicate statistical significance.

Abbreviations: df, degrees of freedom; FDR, False Discovery Rate. control_1000, healthy controls from the 1000 Genomes Project; control_G, control group recruited in the current study. *, P-adjusted ＜0.05. **, P-adjusted ＜0.01.
