# Supplementary material for: AKT1 but not AKT2 single nucleotide polymorphisms are associated with the risk of microscopic polyangiitis
Source: PeerJ. 2026 Feb 16;14:e20791. doi: 10.7717/peerj.20791 (PMC12919311; doi:10.7717/peerj.20791)
Supplement: Supplemental Information 4 — A comprehensive characterization of the SNPs include their genomic locations, predicted functional consequences [file peerj-14-20791-s004.docx]

**Supplemental Table 4** Information of SNPs *in AKT1 and AKT2*

| gene | Chr | SNP | Location(GRCh38) | Functional Consequence |
| --- | --- | --- | --- | --- |
| AKT1 | 14 | rs2498786 | 104796031 | 2KB_upstream_variant, upstream_transcript_variant |
|  |  | rs1130233 | 104773557 | synonymous_variant,coding_sequence_variant |
|  |  | rs2498801 | 104769221 | non_coding_transcript_variant,500B_downstream_variant,  downstream_transcript_variant |
|  |  | rs2494737 | 104779988 | intron_variant |
| AKT2 | 19 | rs7254617 | 40285605 | upstream_transcript_variant,2KB_upstream_variant |
|  |  | rs969531 | 40272959 | intron_variant,genic_upstream_transcript_variant |
|  |  | rs3730051 | 40238790 | intron_variant |

Note: Data was from SNP database of NCBI.
