# Supplementary material for: AKT1 but not AKT2 single nucleotide polymorphisms are associated with the risk of microscopic polyangiitis
Source: PeerJ. 2026 Feb 16;14:e20791. doi: 10.7717/peerj.20791 (PMC12919311; doi:10.7717/peerj.20791)
Supplement: Supplemental Information 5 — Association between the AKT2 genotypes and MPA risk [file peerj-14-20791-s005.docx]

**Supplemental Table 5** Association between the AKT2 genotypes and MPA risk (n=798, adjusted by sex)

| Loci | Model | Genotype | MPA  (n=202) | Control  (n=598) | OR (95% CI) | P-value |
| --- | --- | --- | --- | --- | --- | --- |
| rs7254617 | Codominant | G/G | 157 (77.7%) | 463 (77.8%) | 1.00 | 0.99 |
|  |  | G/A | 41 (20.3%) | 119 (20%) | 1.03 (0.69-1.54) |  |
|  |  | A/A | 4 (2%) | 13 (2.2%) | 0.95 (0.31-2.98) |  |
|  | Dominant | G/G | 157 (77.7%) | 463 (77.8%) | 1.00 | 0.91 |
|  |  | G/A-A/A | 45 (22.3%) | 132 (22.2%) | 1.02 (0.70-1.50) |  |
|  | Recessive | G/G-G/A | 198 (98%) | 582 (97.8%) | 1.00 | 0.93 |
|  |  | A/A | 4 (2%) | 13 (2.2%) | 0.95 (0.30-2.95) |  |
|  | Overdominant | G/G-A/A | 161 (79.7%) | 476 (80%) | 1.00 | 0.88 |
|  |  | G/A | 41 (20.3%) | 119 (20%) | 1.03 (0.69-1.54) |  |
| rs969531 | Codominant | T/T | 97 (48%) | 294 (49.4%) | 1.00 | 0.91 |
|  |  | T/C | 86 (42.6%) | 251 (42.2%) | 1.04 (0.74-1.46) | - |
|  |  | C/C | 19 (9.4%) | 50 (8.4%) | 1.13 (0.63-2.01) |  |
|  | Dominant | T/T | 97 (48%) | 294 (49.4%) | 1.00 | 0.74 |
|  |  | T/C-C/C | 105 (52%) | 301 (50.6%) | 1.06 (0.77-1.45) |  |
|  | Recessive | T/T-T/C | 183 (90.6%) | 545 (91.6%) | 1.00 | 0.72 |
|  |  | C/C | 19 (9.4%) | 50 (8.4%) | 1.11 (0.64-1.93) |  |
|  | Overdominant | T/T-C/C | 116 (57.4%) | 344 (57.8%) | 1.00 | - |
|  |  | T/C | 86 (42.6%) | 251 (42.2%) | 1.02 (0.74-1.41) | 0.90 |
| rs3730051 | Codominant | T/T | 109 (54%) | 338 (56.8%) | 1.00 | - |
|  |  | T/C | 83 (41.1%) | 220 (37%) | 1.17 (0.84-1.63) | 0.50 |
|  |  | C/C | 10 (5%) | 37 (6.2%) | 0.82 (0.39-1.71) |  |
|  | Dominant | T/T | 109 (54%) | 338 (56.8%) | 1.00 | - |
|  |  | T/C-C/C | 93 (46%) | 257 (43.2%) | 1.12 (0.81-1.54) | 0.49 |
|  | Recessive | T/T-T/C | 192 (95%) | 558 (93.8%) | 1.00 | - |
|  |  | C/C | 10 (5%) | 37 (6.2%) | 0.77 (0.37-1.58) | 0.46 |
|  | Overdominant | T/T-C/C | 119 (58.9%) | 375 (63%) | 1.00 | - |
|  |  | T/C | 83 (41.1%) | 220 (37%) | 1.19 (0.86-1.65) | 0.29 |

Note: Analysis was performed by SNPStats ([web tool for SNP analysis](https://www.snpstats.net/start.htm)). P value was adjusted by FDR using the Benjamini-Hochberg procedure. Bolded p-values indicate statistical significance.

Abbreviations: MPA, MPA Group. Control, Control Group.
