# Supplementary material for: AKT1 but not AKT2 single nucleotide polymorphisms are associated with the risk of microscopic polyangiitis
Source: PeerJ. 2026 Feb 16;14:e20791. doi: 10.7717/peerj.20791 (PMC12919311; doi:10.7717/peerj.20791)
Supplement: Supplemental Information 6 — No significant associations were observed for the remaining SNPs ( rs2498801, rs2494737, rs7254617, rs969531 and rs3730051) in any genetic model in blood P_ANCA (+) susceptibility analysis [file peerj-14-20791-s006.docx]

**Supplement Table 6** SNPs (rs2498801, rs2494737, rs7254617, rs969531 and rs3730051) and MPA patients with blood P_ANCA（+）susceptibility analysis (n=737, adjusted by sex)

| Loci | Model | Genotype | ANCA  (n=139) | Control  (n=598) | OR (95% CI) | P-value |
| --- | --- | --- | --- | --- | --- | --- |
| rs2498801 | Codominant | C/C | 68 (48.9%) | 260 (43.7%) | 1.00 | - |
|  |  | T/C | 63 (45.3%) | 269 (45.2%) | 0.94 (0.64-1.38) | 0.13 |
|  |  | T/T | 8 (5.8%) | 66 (11.1%) | 0.47 (0.22-1.04) |  |
|  | Dominant | C/C | 68 (48.9%) | 260 (43.7%) | 1.00 | - |
|  |  | T/C-T/T | 71 (51.1%) | 335 (56.3%) | 0.84 (0.58-1.23) | 0.38 |
|  | Recessive | C/C-T/C | 131 (94.2%) | 529 (88.9%) | 1.00 | - |
|  |  | T/T | 8 (5.8%) | 66 (11.1%) | 0.49 (0.23-1.04) | 0.05 |
|  | Overdominant | C/C-T/T | 76 (54.7%) | 326 (54.8%) | 1.00 | - |
|  |  | T/C | 63 (45.3%) | 269 (45.2%) | 1.05 (0.72-1.53) | 0.80 |
| rs2494737 | Codominant | A/A | 69 (49.6%) | 262 (44%) | 1.00 | - |
|  |  | T/A | 59 (42.5%) | 265 (44.5%) | 0.85 (0.58-1.26) | 0.37 |
|  |  | T/T | 11 (7.9%) | 68 (11.4%) | 0.63 (0.32-1.27) |  |
|  | Dominant | A/A | 69 (49.6%) | 262 (44%) | 1.00 | - |
|  |  | T/A-T/T | 70 (50.4%) | 333 (56%) | 0.81 (0.56-1.17) | 0.27 |
|  | Recessive | A/A-T/A | 128 (92.1%) | 527 (88.6%) | 1.00 | - |
|  |  | T/T | 11 (7.9%) | 68 (11.4%) | 0.68 (0.35-1.33) | 0.25 |
|  | Overdominant | A/A-T/T | 80 (57.5%) | 330 (55.5%) | 1.00 | - |
|  |  | T/A | 59 (42.5%) | 265 (44.5%) | 0.92 (0.63-1.34) | 0.67 |
| rs7254617 | Codominant | G/G | 106 (76.3%) | 463 (77.8%) | 1.00 | - |
|  |  | G/A | 29 (20.9%) | 119 (20%) | 1.08 (0.68-1.72) | 0.80 |
|  |  | A/A | 4 (2.9%) | 13 (2.2%) | 1.44 (0.46-4.54) |  |
|  | Dominant | G/G | 106 (76.3%) | 463 (77.8%) | 1.00 | - |
|  |  | G/A-A/A | 33 (23.7%) | 132 (22.2%) | 1.12 (0.72-1.73) | 0.62 |
|  | Recessive | G/G-G/A | 135 (97.1%) | 582 (97.8%) | 1.00 | - |
|  |  | A/A | 4 (2.9%) | 13 (2.2%) | 1.42 (0.45-4.44) | 0.56 |
|  | Overdominant | G/G-A/A | 110 (79.1%) | 476 (80%) | 1.00 | - |
|  |  | G/A | 29 (20.9%) | 119 (20%) | 1.07 (0.68-1.69) | 0.77 |
| rs969531 | Codominant | T/T | 70 (50.4%) | 294 (49.4%) | 1.00 | - |
|  |  | T/C | 56 (40.3%) | 251 (42.2%) | 0.94 (0.64-1.39) | 0.91 |
|  |  | C/C | 13 (9.3%) | 50 (8.4%) | 1.07 (0.55-2.09) |  |
|  | Dominant | T/T | 70 (50.4%) | 294 (49.4%) | 1.00 | - |
|  |  | T/C-C/C | 69 (49.6%) | 301 (50.6%) | 0.96 (0.66-1.39) | 0.84 |
|  | Recessive | T/T-T/C | 126 (90.7%) | 545 (91.6%) | 1.00 | - |
|  |  | C/C | 13 (9.3%) | 50 (8.4%) | 1.10 (0.58-2.10) | 0.77 |
|  | Overdominant | T/T-C/C | 83 (59.7%) | 344 (57.8%) | 1.00 | - |
|  |  | T/C | 56 (40.3%) | 251 (42.2%) | 0.93 (0.64-1.36) | 0.70 |
| rs3730051 | Codominant | T/T | 78 (56.1%) | 338 (56.8%) | 1.00 | - |
|  |  | T/C | 54 (38.9%) | 220 (37%) | 1.06 (0.72-1.57) | 0.80 |
|  |  | C/C | 7 (5%) | 37 (6.2%) | 0.81 (0.35-1.88) |  |
|  | Dominant | T/T | 78 (56.1%) | 338 (56.8%) | 1.00 | - |
|  |  | T/C-C/C | 61 (43.9%) | 257 (43.2%) | 1.03 (0.71-1.49) | 0.89 |
|  | Recessive | T/T-T/C | 132 (95%) | 558 (93.8%) | 1.00 | - |
|  |  | C/C | 7 (5%) | 37 (6.2%) | 0.79 (0.34-1.80) | 0.56 |
|  | Overdominant | T/T-C/C | 85 (61.1%) | 375 (63%) | 1.00 | - |
|  |  | T/C | 54 (38.9%) | 220 (37%) | 1.09 (0.74-1.59) | 0.67 |

Note: Analysis was performed by SNPStats ([web tool for SNP analysis](https://www.snpstats.net/start.htm)).

Abbreviations: ANCA, MPA patients with blood P_ANCA（+）Group. Control, Control Group.
