# Supplementary material for: AKT1 but not AKT2 single nucleotide polymorphisms are associated with the risk of microscopic polyangiitis
Source: PeerJ. 2026 Feb 16;14:e20791. doi: 10.7717/peerj.20791 (PMC12919311; doi:10.7717/peerj.20791)
Supplement: Supplemental Information 7 — the AKT1 gene block to be independently associated with MPA susceptibility [file peerj-14-20791-s007.docx]

**Supplement Table 7** Assessing the Collective Association of the AKT1 Gene Block with MPA Risk adjusted by sex

| Model Comparison | -2 Log Likelihood | df | χ² | P-value |
| --- | --- | --- | --- | --- |
| Reduced Model (AKT2 SNPs only) | 451.36 | 4 | - | - |
| Full Model (AKT1 + AKT2 SNPs) | 438.92 | 8 | - | - |
| Likelihood-Ratio Test (Full vs. Reduced) | - | 4 | 24.885 | **5.31×10⁻⁵***** |

Note: Analyses were performed using multivariable logistic regression. The sample size for all models was 798 participants. Bolded p-values indicate statistical significance.

Abbreviations: df, degrees of freedom. *******, P-value ＜0.001.
