# Supplementary material for: AKT1 but not AKT2 single nucleotide polymorphisms are associated with the risk of microscopic polyangiitis
Source: PeerJ. 2026 Feb 16;14:e20791. doi: 10.7717/peerj.20791 (PMC12919311; doi:10.7717/peerj.20791)
Supplement: Supplemental Information 8 — A significant association between the AKT1 gene set (4 SNPs) and decreased MPA risk. [file peerj-14-20791-s008.docx]

**Supplement Table 8** Association of AKT1 and AKT2 Gene Sets with MPA Risk adjusted by sex

| Gene Set | No. of SNPs | β Coefficient | Odds Ratio (95% CI) | P-value | P-adjusted |
| --- | --- | --- | --- | --- | --- |
| AKT1 | 4 | -0.123 | 0.884 (0.820 - 0.953) | **0.002** | **0.004**** |
| AKT2 | 3 | 0.028 | 1.028 (0.902 - 1.171) | 0.686 | 0.686 |

Note: Analysis were performed using Plink (v1.9). P value was adjusted by FDR using the Benjamini-Hochberg procedure. Bolded p-values indicate statistical significance.

Abbreviations: CI, confidence interval. **, P-adjusted ＜0.01.
