# Supplementary material for: AKT1 but not AKT2 single nucleotide polymorphisms are associated with the risk of microscopic polyangiitis
Source: PeerJ. 2026 Feb 16;14:e20791. doi: 10.7717/peerj.20791 (PMC12919311; doi:10.7717/peerj.20791)
Supplement: Supplemental Information 9 [file peerj-14-20791-s009.docx]

**Supplement Table 7** SNP-SNP interaction analysis performed by GMDR

| Best model | Training Bal. Acc. | Testing Bal. Acc. | CV | P-value (Sign Test) |
| --- | --- | --- | --- | --- |
| X1 | 0.5831 | 0.5872 | 10/10 | 0.0107(9) |
| X2/ X3 | 0.5838 | 0.5862 | 6/10 | 0.0107(9) |
| **X2/ X3/ X4** | **0.6026** | **0.5805** | **10/10** | **0.0010(10) ^**^** |
| X2/ X3/X4/X5 | 0.6106 | 0.5600 | 10/10 | 0.0107(9) ^*^ |
| X1/X2/ X3/X4/X5 | 0.6110 | 0.5600 | 10/10 | 0.0107(9) ^*^ |

Note: Analysis was performed by GMDR (version [0.9](https://www.snpstats.net/start.htm)). X1, rs5811155. X2, rs2498786 X3, rs2494752. X4, rs1130233. X5, rs2498801.

Abbreviations: SNP: single nucleotide polymorphism; GMDR: generalized multifactor- dimensionality reduction; Training Bal. Acc.: training balance accuracy; Testing Bal. Acc.: testing balance accuracy; CV: cross-validation consistency.
