# Supplementary figures and images for: AKT1 but not AKT2 single nucleotide polymorphisms are associated with the risk of microscopic polyangiitis
Source: PeerJ. 2026 Feb 16;14:e20791. doi: 10.7717/peerj.20791 (PMC12919311; doi:10.7717/peerj.20791)

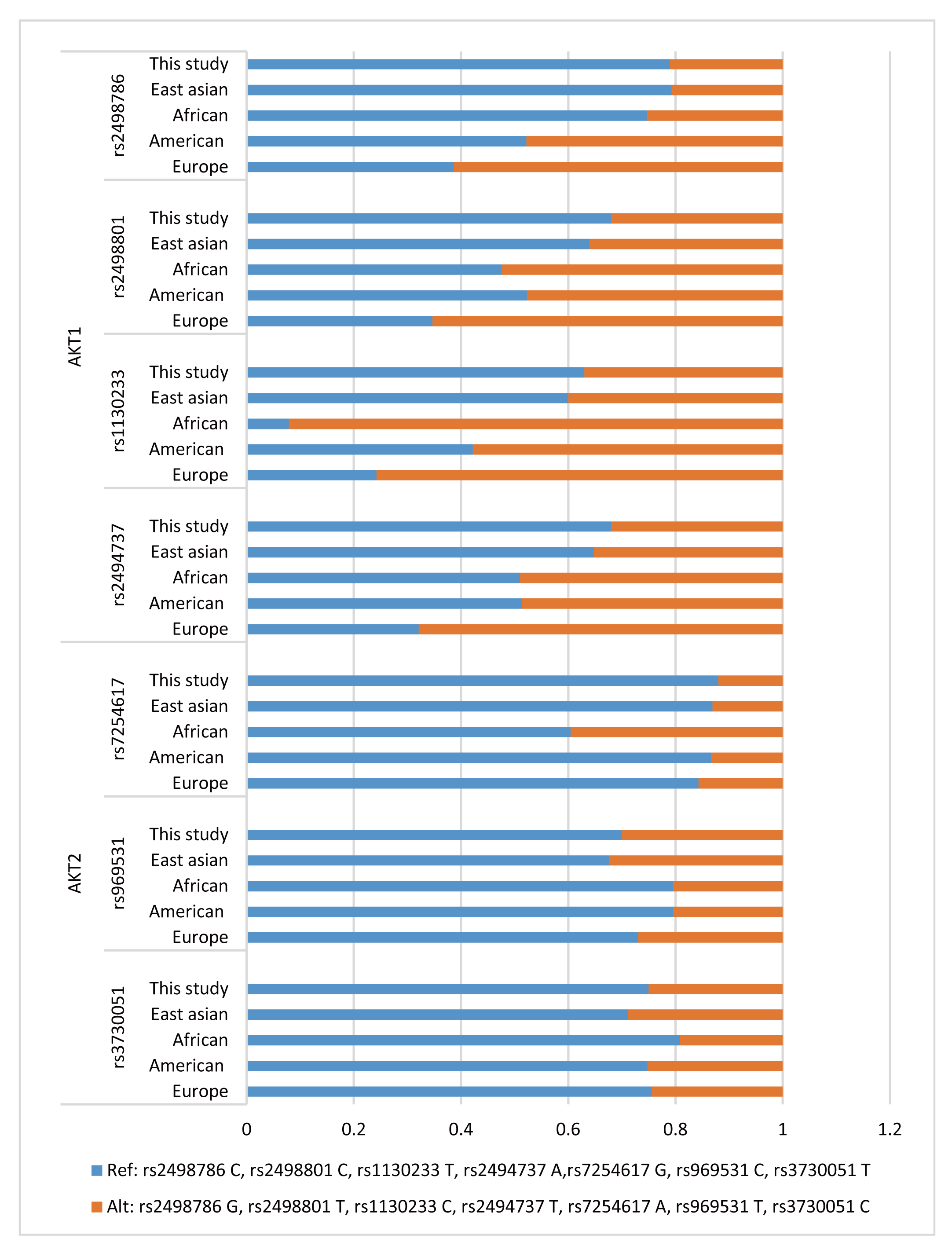

Supplement: Supplemental Information 11 — The allelic frequency distribution across major populations (East Asian, African, Amerindian, and European) in both the 1000 Genomes Project and this study cohort.Data for the 1000 Genomes populations were sourced from publicly available repositories hosted by the National Center for Biotechnology Information (NCBI; https://www.ncbi.nlm.nih.gov/) and the International Genome Sample Resource (IGSR; https://www.internationalgenome.org/). [file peerj-14-20791-s011.png]
