# Supplementary material for: AKT1 but not AKT2 single nucleotide polymorphisms are associated with the risk of microscopic polyangiitis
Source: PeerJ. 2026 Feb 16;14:e20791. doi: 10.7717/peerj.20791 (PMC12919311; doi:10.7717/peerj.20791)
Supplement: Supplemental Information 12 [file peerj-14-20791-s012.pdf]

**Sample population URL：**
https://www.internationalgenome.org/data-portal/sample

**Data collections：**
https://www.internationalgenome.org/data-portal/data-collection/phase-3

**For downstream analyses, VCF files were converted to PED/MAP format using the VCF to PED Converter provided by IGSR:**
**https://grch37.ensembl.org/Multi/Tools/VcftoPed**

**File URL：**
AKT1rs2498786
https://ftp.1000genomes.ebi.ac.uk/vol1/ftp/release/20130502/ALL.chr14.phase3_shapeit2_mvncall_integrated_v5b.20130502.genotypes.vcf.gz

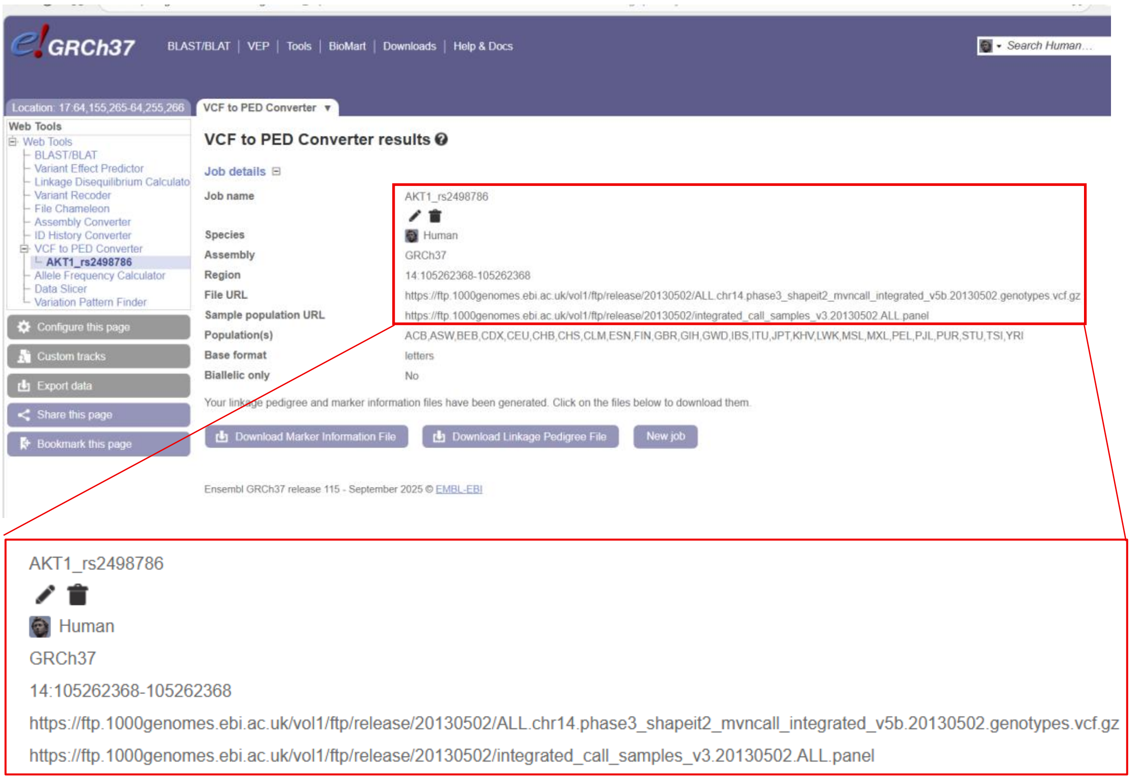


AKT1rs2498801
https://ftp.1000genomes.ebi.ac.uk/vol1/ftp/release/20130502/ALL.chr14.phase3_shapeit2_mvncall_integrated_v5b.20130502.genotypes.vcf.gz

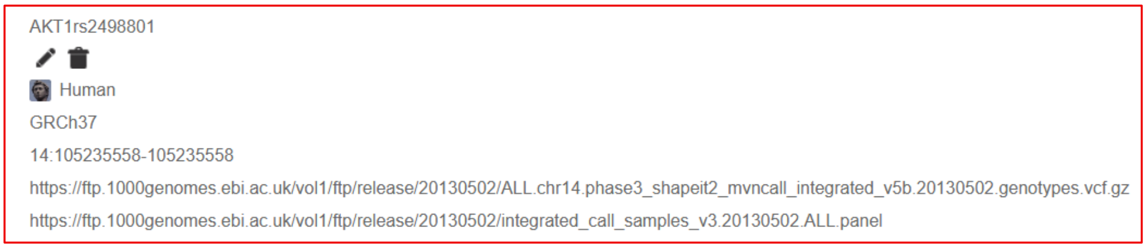


AKT1rs1130233

https://ftp.1000genomes.ebi.ac.uk/vol1/ftp/release/20130502/ALL.chr14.phase3_shapeit2_mvncall_integrated_v5b.20130502.genotypes.vcf.gz

AKT1rs1130233
Human
GRCh37
14:105239894-105239894
https://ftp.1000genomes.ebi.ac.uk/vol1/ftp/release/20130502/ALL.chr14.phase3_shapeit2_mvncall_integrated_v5b.20130502.genotypes.vcf.gz
https://ftp.1000genomes.ebi.ac.uk/vol1/ftp/release/20130502/integrated_call_samples_v3.20130502.ALL.panel

AKT1rs2494737

https://ftp.1000genomes.ebi.ac.uk/vol1/ftp/release/20130502/ALL.chr14.phase3_shapeit2_mvncall_integrated_v5b.20130502.genotypes.vcf.gz

AKT1rs2494737
Human
GRCh37
14:105246325-105246325
https://ftp.1000genomes.ebi.ac.uk/vol1/ftp/release/20130502/ALL.chr14.phase3_shapeit2_mvncall_integrated_v5b.20130502.genotypes.vcf.gz
https://ftp.1000genomes.ebi.ac.uk/vol1/ftp/release/20130502/integrated_call_samples_v3.20130502.ALL.panel

AKT2rs7254617

https://ftp.1000genomes.ebi.ac.uk/vol1/ftp/release/20130502/ALL.chr19.phase3_shapeit2_mvncall_integrated_v5b.20130502.genotypes.vcf.gz

AKT2rs7254617
Human
GRCh37
19:40791512-40791512
https://ftp.1000genomes.ebi.ac.uk/vol1/ftp/release/20130502/ALL.chr19.phase3_shapeit2_mvncall_integrated_v5b.20130502.genotypes.vcf.gz
https://ftp.1000genomes.ebi.ac.uk/vol1/ftp/release/20130502/integrated_call_samples_v3.20130502.ALL.panel

AKT2rs969531

https://ftp.1000genomes.ebi.ac.uk/vol1/ftp/release/20130502/ALL.chr19.phase3_shapeit2_mvncall_integrated_v5b.20130502.genotypes.vcf.gz

AKT2rs969531
Human
GRCh37
19:40778866-40778866
https://ftp.1000genomes.ebi.ac.uk/vol1/ftp/release/20130502/ALL.chr19.phase3_shapeit2_mvncall_integrated_v5b.20130502.genotypes.vcf.gz
https://ftp.1000genomes.ebi.ac.uk/vol1/ftp/release/20130502/integrated_call_samples_v3.20130502.ALL.panel

AKT2rs3730051

https://ftp.1000genomes.ebi.ac.uk/vol1/ftp/release/20130502/ALL.chr19.phase3_shapeit2_mvncall_integrated_v5b.20130502.genotypes.vcf.gz

AKT2rs3730051
Human
GRCh37
19:40744697-40744697
https://ftp.1000genomes.ebi.ac.uk/vol1/ftp/release/20130502/ALL.chr19.phase3_shapeit2_mvncall_integrated_v5b.20130502.genotypes.vcf.gz
https://ftp.1000genomes.ebi.ac.uk/vol1/ftp/release/20130502/integrated_call_samples_v3.20130502.ALL.panel
